# Suppression of tumor immune microenvironment via microRNA‐1 after epidermal growth factor receptor‐tyrosine kinase inhibitor resistance acquirement in lung adenocarcinoma

**DOI:** 10.1002/cam4.3639

**Published:** 2020-12-11

**Authors:** Sachiko Kawana, Ryoko Saito, Yasuhiro Miki, Yuichiro Kimura, Jiro Abe, Ikuro Sato, Mareyuki Endo, Shunichi Sugawara, Hironobu Sasano

**Affiliations:** ^1^ Department of Pathology Tohoku University School of Medicine Miyagi Japan; ^2^ Department of Respiratory Medicine Sendai Kousei Hospital Miyagi Japan; ^3^ Department of Thoracic Surgery Miyagi Cancer Center Miyagi Japan; ^4^ Department of Pathology Miyagi Cancer Center Miyagi Japan; ^5^ Department of Pathology Sendai Kousei Hospital Miyagi Japan

**Keywords:** EGFR‐TKI resistance, immunotherapy predictor, miR‐1, tumor immune microenvironment

## Abstract

Immunotherapy is considered one of the most important therapeutic strategies for patients with lung adenocarcinoma after the development of epidermal growth factor receptor tyrosine kinase inhibitor (EGFR‐TKI) resistance. However, useful predictors of immunotherapy for these patients has not been examined well, although the status of the tumor immune microenvironment (TIME), including programmed death‐ligand 1 expression and lymphocyte infiltration, has been generally known to provide predictive markers for the efficacy of immunotherapy. This study aimed to clarify novel predictors of immunotherapy following EGFR‐TKI resistance in lung adenocarcinoma, especially regarding micro RNA (miRNA). We evaluated the correlation between EGFR‐TKI resistance and lymphocyte infiltration, before and after acquiring EGFR‐TKI resistance, in 21 cases of lung adenocarcinoma, and further explored this by *in vitro* studies, using miRNA PCR arrays. Subsequently, we transfected miRNA‐1 (miR‐1), the most variable miRNA in this array, into three kinds of lung cancer cells, and examined the effects of miR‐1 on EGFR‐TKI sensitivity, cytokine expression and lymphocyte migration. Histopathological examination demonstrated that infiltration levels of CD8‐positive T cells were significantly decreased after development of EGFR‐TKI resistance. *In vitro* studies revealed that miR‐1 significantly inhibited EGFR‐TKI effect and induction of cytokines, such as C‐C motif chemokine ligand 5 and C‐X‐C motif chemokine ligand 10, causing inhibition of monocyte migration. These results indicate that the upregulated miR‐1 might suppress the TIME, following development of EGFR‐TKI resistance. Therefore, miR‐1 could be a clinically useful marker to predict therapeutic efficacy of immunotherapy in lung adenocarcinoma patients with EGFR‐TKI resistance.

## Graphical abstract

We firstly revealed that intra‐tumoral CD8‐positive T cells are decreased in lung adenocarcinoma patients acquiring EGFR‐TKI resistance when levels of miR‐1 are raised. In addition, patients with high expression of miR‐1 may be less likely to benefit from subsequent immunotherapy using immune checkpoint inhibitor (ICI). Therefore, an evaluation of serum miR‐1, before treatment and after acquiring EGFR‐TKI resistance, could contribute to predicting therapeutic efficacy of subsequent ICI therapy for the patient.

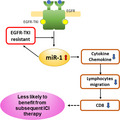

## 1 BACKGROUND

Lung cancer is a leading cause of cancer‐related deaths, worldwide.^1^, ^2^ In non‐small cell lung cancer (NSCLC), especially in adenocarcinoma, “driver mutations” have been discovered in genes such as *EGFR* (epidermal growth factor receptor), which are important to the processes of cancerization and proliferation.^3^, ^4^, ^5^ In East Asian studies, approximately half of the adenocarcinoma patients were found to harbor EGFR mutations.^6^ In addition, according to current guidelines for clinical practice, EGFR‐tyrosine kinase inhibitor (TKI) is recommended as the first choice of therapy in the patients with advanced‐stage NSCLC, harboring *EGFR* mutations.^7^, ^8^, ^9^ EGFR‐TKI exerts reasonable therapeutic effects upon EGFR‐mutation‐positive NSCLC patients^10^, ^11^, ^12^, ^13^, ^14^, ^15^, ^16^ but, in many cases, the development of therapeutic resistance is inevitable within several months, due to the newly acquired T790M mutation, and others.^17^, ^18^ This is presently conceived as one of the most serious clinical problems of this particular therapy. On the other hand, an immune checkpoint inhibitor (ICI) has attracted great interest as a new treatment strategy for lung cancer patients and has been approved in EGFR‐TKI‐resistant NSCLC patients. In addition, immunohistochemical evaluation of programmed death‐ligand 1 (PD‐L1) in tumor cells is currently approved as a potential biomarker for predicting the clinical efficacy of ICI, by such as anti‐ programmed death 1 (PD‐1) antibodies.^19^ However, it is also true that the overall response rate to ICI in NSCLC is only about 20%, at most,^20^, ^21^ and 40%–50% in those with relatively abundant expression of PD‐L1 in carcinoma cells.^22^ In working toward solving these clinical problems, novel predictors of ICI therapy, other than the immunohistochemical status of PD‐L1, have been explored. Among these, ICIs were reported to be more effective in the patients with abundant intra‐tumoral infiltration of CD8+ T lymphocytes in lung cancer tissues, and these patients generally had good prognoses.^23^, ^24^ In addition, therapeutic effects of ICI have been reported to be poor in EGFR mutation‐positive cases,^21^, ^25^ and there are some previous reports showing a correlation between EGFR‐TKI resistance and poor benefit of ICI.^26^ The administration of EGFR‐TKI itself has been reported to change the tumor immune microenvironment (TIME), including reduced PD‐L1 expression and forkhead box P3 (Foxp3)‐positive T cell reduction,^26^, ^27^, ^28^, ^29^ and this change in PD‐L1 expression has been cited as a reason for the poor effectiveness of ICI after the acquirement of EGFR‐TKI resistance.^27^, ^30^ However, the relationship between changes in TIME and the mechanism of EGFR‐TKI resistance in detail has not been examined yet.

Therefore, we hypothesized that the acquisition of EGFR‐TKI resistance in carcinoma cells could alter the therapeutic efficacy of ICI through changing aspects of the TIME, such as the level of lymphocyte infiltration in NSCLC patients, based on the studies reported above. We also explored the status of microRNAs (miRNAs) in order to further elucidate its mechanisms, because miRNAs are well known to be involved in tumor development and progression^31^, ^32^, ^33^ and in the EGFR signaling pathway and cytokine expression in lung carcinoma.^34^ However, to the best of our knowledge, there have been few reports of a possible correlation between miRNAs and ICI in lung cancer.

This is the first study to examine the correlation between EGFR‐TKI resistance and TIME, with particular emphasis on changes in miRNAs. Recently, ICI has become one of the main therapeutic methods after acquiring EGFR‐TKI resistance, and it is considered to be important to study the relationship between them. In particular, we aimed to explore new predictors of therapeutic efficacy and the therapeutic targets of ICI in patients with EGFR‐TKI‐resistant lung adenocarcinoma.

## 2 MATERIALS AND METHODS

### 2.1 Patient information

A total of 119 cases were examined in this study in order to explore changes in the TIME before and after developing EGFR‐TKI resistance. NSCLC specimens were obtained from Japanese patients who developed therapeutic resistance to EGFR‐TKI therapy from 2010 to 2017 at Sendai Kosei Hospital, Tohoku University Hospital, and Miyagi Cancer Center. Of 119 cases, paired histological specimens of pre‐ and post‐EGFR‐TKI treatment were retrieved from 21 cases (totaling 42 samples; 36 samples: biopsy, six samples: surgical resection). All of these cases were histologically diagnosed as adenocarcinoma, seven male and 14 female, median age 73 years (range 56−87 years, standard deviation 7.2 years). Our cases included all major types of mutation of EGFR, including exon 19 deletion in nine cases, and L858R. Ninety‐eight cases were not examined because either or both paired samples corresponded to lymph nodes or liquid samples such as pleural effusion, in which we could not evaluate the status of TIME. We could only evaluate the status of EGFR mutation in 10 of 21 specimens from the samples after acquiring EGFR‐TKI resistance. As a result, five out of 10 cases were found to be positive for T790M (Table 1). The primary treatment was EGFR‐TKI alone in 15 cases, chemotherapy (Carboplatin, Pemetrexed) combined with EGFR‐TKI in three cases, where EGFR‐TKI was used as the second‐line treatment alone, or in combination with chemotherapy. Chemotherapy (Cisplatin, Carboplatin, Pemetrexed, Vinorelbine, Paclitaxel) was used alone in three cases. Carboplatin and Pemetrexed were the main drugs used for chemotherapy. All treatment was performed according to current clinical practice guidelines for treating lung cancer.

**TABLE 1:** Clinicopathological characteristics of the patients

	n = 21
Age	73 ± 7.2 (56–87)
Sex	
Male	7
Female	14
Smoking status	
Non‐smoker	14
Smoker	6
Unknown	1
Brinkman Index	195.8 ± 403
Histology	
Adenocarcinoma	21
EGFR mutation	
Exon 19 deletion	9
L858R	12
T790M (before EGFR‐TKI resistance acquirement)	0
T790M (after EGFR‐TKI resistance acquirement)	5*

Data are presented as mean ± SD. Brinkman Index was calculated by the number of cigarettes smoked per day multiplied by the number of years of smoking history of individuals.

Abbreviations: EGFR, epidermal growth factor receptor; EGFR‐TKI, EGFR tyrosine kinase inhibitor.

* We could only evaluate the status of EGFR mutation in 10 of 21 specimens after EGFR‐TKI resistance acquirement.

All specimens had been fixed in 10% formalin and embedded in paraffin. The study protocol was approved by the Ethics Committee at the Tohoku University School of Medicine (2018–1–612), Sendai Kousei Hospital (29–16) and Miyagi Cancer Center (2018–25–1).

### 2.2 Immunohistochemistry

The characteristics of the primary antibodies used in this study were as follows: CD3 ([Dako, Glostrup, Denmark], Mouse monoclonal, 1:500, Autoclave in citrate buffer), CD4 ([Nichirei, Tokyo, Japan], Mouse monoclonal, 1:1, autoclaved in citrate buffer (pH 9)), CD8 ([Dako], Mouse monoclonal, 1:50, autoclaved in citrate buffer), FoxP3 ([Abcam], Mouse monoclonal, 1:100, autoclaved in citrate buffer). A Histofine Kit (Nichirei) using the streptavidin‐biotin amplification method was used. The antigen‐antibody complex was visualized using the 3,3ʹ‐diaminobenzidine (DAB) solution (1 mM DAB, 50 mM Tris‐HCL buffer, pH 7.6, and 0.006% H_2_O_2_) and counterstained with hematoxylin.

### 2.3 Histopathological evaluation

The stained slides were evaluated independently by two of the authors (SK and RS). We examined a whole slide for each individual case. The cells demonstrating higher immunointensity than the background were tentatively defined as “positive” in this study. Immunoreactivity of CD3, CD4, and CD8 was all detected in the cell membrane, that of Foxp3 in the nucleus of lymphocytes. The immunopositive rate for CD3 was used as a measure of infiltration level when we evaluated CD4, CD8 and Foxp3.

### 2.4 Cell culture

The following cell lines were used in this study: PC9 (Riken Cell Bank, Tsukuba, Japan) and NCI‐H1975 (H1975) (American Type Cell Culture Collection (ATCC), Manassas, VA, USA) for lung adenocarcinoma; peripheral blood mononuclear cells (PBMCs) from a healthy donor (Precision Bioservices, Frederick, MD, USA). PC9/6M and PC9/ER (erlotinib‐resistant cell line) were established in our laboratory by methods reported previously.^35^ PC9 (Immuno‐biological Laboratories) was exposed to erlotinib and the concentration was gradually increased at 2‐month intervals as follows: 10 nM, 1 μM and 5 μM. PC9 cells were also cultured for 6 months (PC9/6M) without erlotinib, to eliminate the effects of long‐term cell culture. The EGFR mutation profiles of these cell lines were as follows: PC9 and PC9/6M; exon 19 deletion, PC9/ER; exon 19 deletion, L858R mutation, and T790M mutation, H1975; L858R mutation and T790M mutation.

These cells were maintained in Roswell Park Memorial Institute (RPMI) medium 1640 (Sigma‐Aldrich, St. Louis, MO, USA) supplemented with 10% fetal bovine serum (FBS) (Nichirei) and 1% penicillin/streptomycin at 37°C in a humidified incubator containing 5% CO_2_.

### 2.5 miRNA polymerase chain reaction (PCR) array

Total RNA was extracted from PC9/6M and PC9/ER using the TRI reagent (Molecular Research Center, Cincinnati, OH, USA). The cDNA synthesis was performed using 1000 ng of total RNA, based on the recommended protocol, using the miScript HiSpec Buffer (Qiagen, Hilden, Germany) (37°C for 60 min and then at 95°C for 5 min). After the dilution of cDNA, PCR array was performed based on a SybrGreen protocol and using human tumor suppressor gene miScript miRNA PCR array (MIHS‐119Z) (Qiagen) following the manufacturer's protocol. These arrays contain the probes for 92 miRNAs whose expression has been known to be altered in human cancer. The quantitative real‐time PCR reaction was performed in the 7500 Fast Real‐Time PCR system (Applied Biosystems, Foster City, CA, USA) following the program: 95°C for 15 min and then 40 cycles of 94°C for 15 s, 55°C for 30 s and 70°C for 30 s. The analysis of miRNA expression was performed using the data analysis portal provided by Qiagen (https://dataanalysis.qiagen.com/mirna/arrayanalysis.php).

### 2.6 miR‐1 transfection

In order to further evaluate the effects of miR‐1 on tumor cells, PC9 (2.0 × 10^5^ cells/mL), PC9/ER (1.0 × 10^5^ cells/mL) and H1975 (1.0 × 10^5^ cells/mL) were each treated with 50 nM of miR‐1 (Bioneer, Daejeon, Korea) for 24, 48, or 72 h. The sequence of miR‐1 used was as follows: miR‐1; 5ʹ‐ACAUACUUCUUUAUAUGCCCAU‐3ʹ. AccuTarget™ miRNA mimic Negative Control (Bioneer) was used as a negative control. Transfection was performed according to the manufacturer's protocol.

### 2.7 Cytokine antibody array

In this study, we treated PC9 (2.0 × 10^5^ cells/mL) with 50 nM miR‐1 for 24 h in the RPMI 1640 medium containing 10% FBS in order to further assess the potential effects of miR‐1 on secretion levels of cytokines. The medium was subsequently removed. The cells were then washed with phosphate‐buffered saline (PBS) and incubated in phenol red‐ and FBS‐free RPMI 1640 medium for 24 h. The conditioned medium was used in samples. We used the Human Cytokine Antibody Array 5 (RayBiotech, Peachtree Corners, GA, USA), which can detect 80 cytokines. The membranes were then spotted with cytokine‐specific antibodies and were analyzed. The signal was detected using the Image Lab TM software (BIO‐RAD Laboratories, Inc., Hercules, CA, USA).

### 2.8 Cell viability assays

To assess the effect of miR‐1 on EGFR‐TKI sensitivity, PC9 (5000 cells/well) was seeded into 96‐well plates and incubated in RPMI‐1640 medium containing 10% FBS for several hours before transfection. After preincubation, the cells were transfected with miR‐1 or miRNA negative control. After 24 h of transfection, the medium was removed and each cell was treated with 5 µM gefitinib (Biaffin, Kassel, Germany) or 5 µM erlotinib (Roche Diagnostics, Mannheim, Germany). At 0, 24, 48, and 72 h after the treatment, the cell numbers were evaluated using a Cell Counting Kit‐8 (Dojindo, Kumamoto, Japan).

### 2.9 Real‐time RT‐PCR

Total RNA was extracted carefully from the cells, using the TRI reagent (Molecular Research Center) and was reverse transcribed to cDNA using a QuantiTect Reverse Transcription Kit (Qiagen). The levels of mRNA expression were semi‐quantified using a real‐time RT‐PCR in a LightCycler System (Roche Diagnostics). The PCR mixture (20 μL) included 1.0‐μM primer and 2× QuantiTect SYBR Green PCR Master Mix (Qiagen). The following PCR protocol was used in this study: initial denaturation at 95°C for 5 min, followed by 40 amplification cycles of 95°C for 10 s and annealing at 60°C for 30 s. The primers used for PCR included: C‐C Motif Chemokine Ligand 5 (CCL5) forward, 5ʹ‐CAGTCGTCTTTGTCACCCGA‐3ʹ; CCL5 reverse, 5ʹ‐GAGCAAGCAGAAACAGGCAAA‐3ʹ; C‐X‐C Motif Chemokine Ligand 10 (CXCL10) forward, 5ʹ‐AGCAGTTAGCAAGGAAAGGTCTAA‐3ʹ; CXCL10 reverse, 5ʹ‐TGTGTGGTCCATCCTTGGAA‐3ʹ; Interleukin (IL)‐8 forward, 5ʹ‐AGGAGTGCTAAAGAACTTAGATGTCAGTGC‐3ʹ; IL‐8 reverse, 5ʹ‐GTGGTCCACTCTCAATCACTCTCAGTTC‐3ʹ; IL‐6 forward, 5ʹ‐TCATCTCATTCTGCGCAGCTTTAAGGAG‐3ʹ; IL‐6 reverse, 5ʹ‐ATGCCCATTAACAACAATCTGAGGTG‐3ʹ; Tumor necrosis factor (TNF)α forward, 5ʹ‐ CTTCAGACACCCTCAACCTCTT‐3ʹ; TNFα reverse, 5ʹ‐CACATTCCTGAATCCCAGGT‐3ʹ; Ribosomal protein L13a (RPL13A) forward, 5ʹ‐CCTGGAGGAGAAGAGGAAAG‐3ʹ; and RPL13A reverse, 5ʹ‐TTGAGGACCTCTGTGTATTT‐3ʹ. The mRNA levels of *CCL5*, *CXCL10*, *IL*‐*8*, *IL*‐*6*, and *TNFα* genes were expressed as ratios of the RPL13A mRNA level.

### 2.10 PBMC migration assay

We performed migration assays using a Chemotaxicell chamber containing membranes with 5‐μm pore size (Kurabo, Osaka, Japan) and 24‐well plates. After treating PC9 (2.0 × 10^5^ cells/ml) with 50‐nM miR‐1 for 24 h, we washed the cells with PBS and exchanged the medium to phenol red‐ and FBS‐free RPMI 1640 medium. After 24 h of incubation, we used this as a conditioned medium in the lower chambers. The PBMCs were then plated in the upper chambers (4.5 × 10^4^ cells/well) in phenol red‐ and FBS‐free RPMI 1640 medium. After a further 24 h of incubation, the number of migrated cells in the lower chamber was counted using a TC 20TM Automated Cell Counter (Bio‐Rad Laboratories, Inc.).

### 2.11 Statistical analysis

Statistical analysis was performed using JMP Pro 14.0.0 (SAS Institute, Cary, NC, USA). Statistical differences between the two groups and paired analyses were evaluated by the Student t‐test. Statistical significance was defined as *p* < 0.05 in this study.

## 3 RESULTS

### 3.1 The changes to CD8‐positive T lymphocyte infiltration after EGFR‐TKI resistance acquirement

**FIGURE 1:**
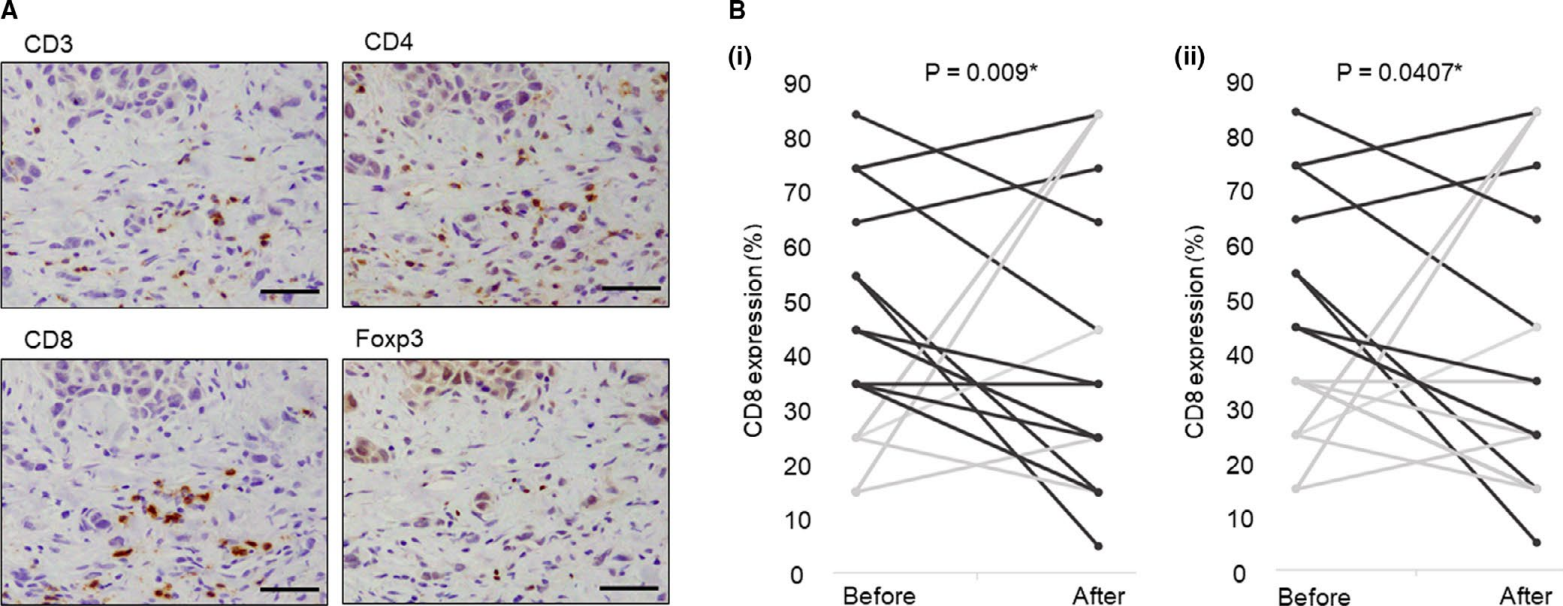
Changes of tumor tissue microenvironment, before and after acquiring EGFR‐TKI resistance, in human lung cancer tissues. A, Representative findings of immunohistochemistry of CD3, CD4, CD8 and Foxp3 in human lung cancer tissue (bar: 50 µm). Immunoreactivity of CD3, CD4, and CD8 was detected in the cell membrane, and that of Foxp3 was in nuclei of carcinoma cells. B, Paired analyses of CD8 changed before and after acquiring EGFR‐TKI resistance. When limited to cases showing over 30% (i) or 40% (ii) of CD8‐positive rate before EGFR‐TKI treatment (bold line), the rate significantly decreased following EGFR‐TKI treatment

We compared the changes in tumor infiltration levels of CD4, CD8, or Foxp3‐positive T cells in tumor tissues before and after EGFR‐TKI treatment, in the patients who developed EGFR‐TKI resistance. Representative findings are summarized in Figure 1A. There were no significant differences in the overall CD8‐positive rate (*p* = 0.669) but, when limited to the cases harboring more than 30% or 40% of CD8‐positive rate before EGFR‐TKI treatment, the rate significantly decreased following the EGFR‐TKI treatment (30%: *p* = 0.009, 40%: *p* = 0.0407) (Figure 1B). There were no significant changes in CD4, FoxP3‐positive, and CD4/8 ratio (*p* = 0.5998, *p* = 0.5471, *p* = 0.5383, respectively) before and after the therapy.

### 3.2 Comprehensive evaluation of miRNA expression before and after EGFR‐TKI resistance acquirement

We subsequently performed the miRNA PCR array to explore the change of miRNA expression following acquired EGFR‐TKI resistance. Fold change of expression levels for each miRNA in PC9/ER (an EGFR‐TKI resistant cell line) from those in PC9/6M (an EGFR‐TKI sensitive cell line) was evaluated in this study (n = 3). Seven miRNAs, whose expression levels had more than twofold changes, were summarized in Table 2. Among these, miR‐1 demonstrated the largest increase (fold change: 8.42, *p* = 0.00014).

**TABLE 2:** Result of miRNA PCR array

miRNA	PC9/ER (fold change compared with PC9/6M)	*p* value
Let−7b	2.01	0.01995*
miR−1	8.42	0.00014*
miR−140	5.15	0.00054*
miR−142	6.13	0.00375*
miR−143	2.85	0.04118*
miR−146a	8.09	<0.0001*
miR−152	3.02	0.00034*

PCR, polymerase chain reaction.

* *p* value < 0.05, significant.

### 3.3 MiR‐1 reduced the effects of EGFR‐TKIs

**FIGURE 2:**
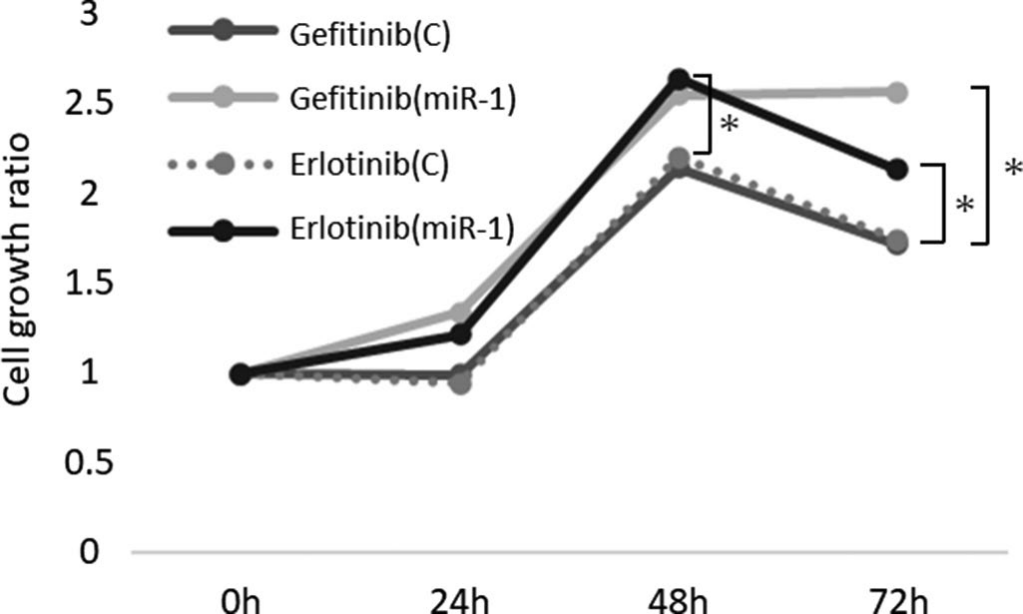
MiR‐1 reduced the effects of EGFR‐TKIs. Results of cell viability assay (n = 6). In PC9, significant cell proliferation was observed in the miR‐1 transfection group at 48 and 72 h after treatment with gefitinib or erlotinib (gefitinib/erlotinib: after 48 h; *p* = 0.0002/*p* = 0.0001, after 72 h; *p* = 0.0002/*p* = 0.0291)

MiR‐1 transfection significantly reduced the sensitivity for gefitinib or erlotinib at 48 and 72 h after the treatment (gefitinib/erlotinib: after 48 h; *p* = 0.0002/*p* = 0.0001, after 72 h; *p* = 0.0002/*p* = 0.0291) (Figure 2).

### 3.4 MiR‐1 inhibited cytokine expressions and monocytes migration

**FIGURE 3:**
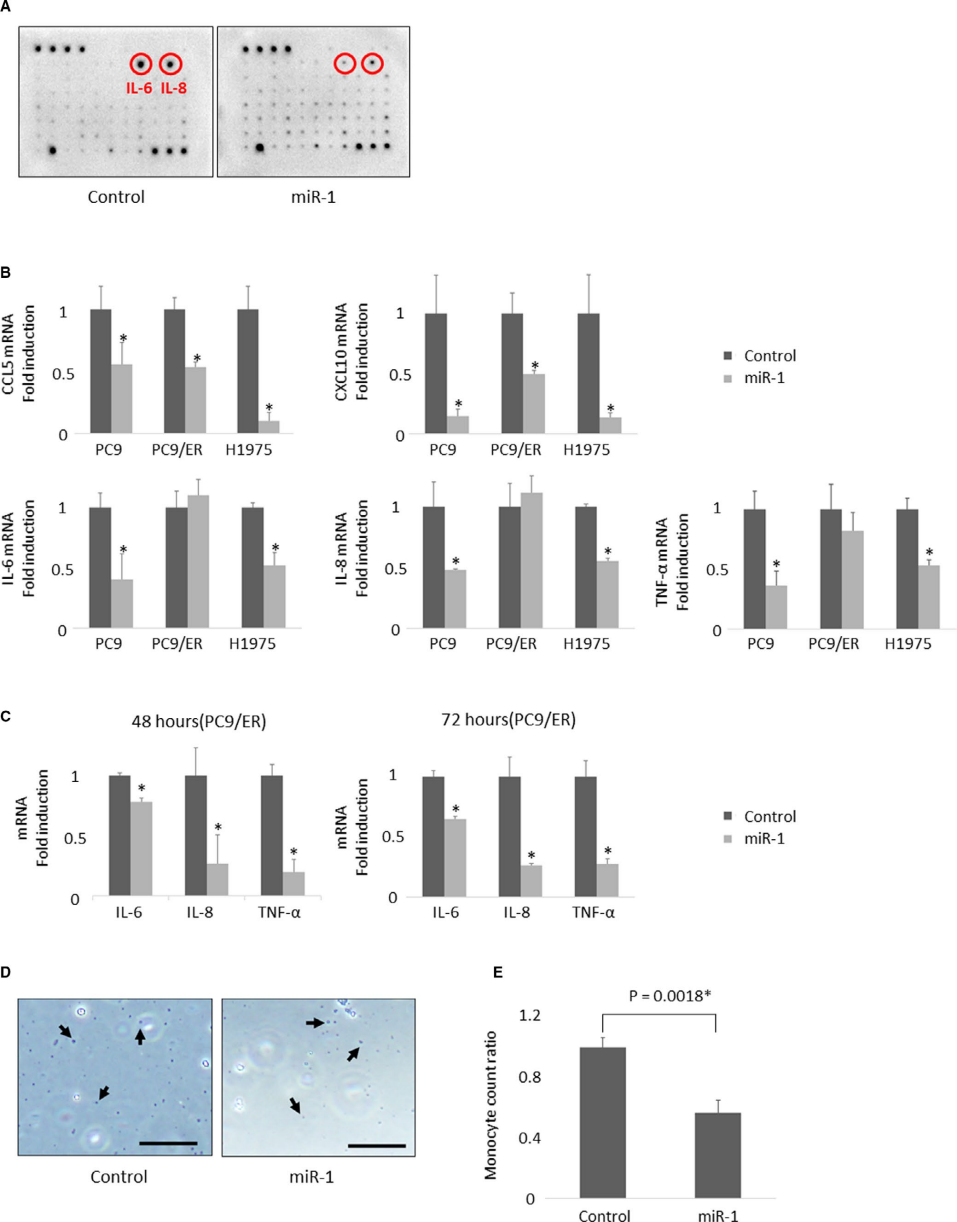
Changes in tumor tissue microenvironment before and after acquiring EGFR‐TKI resistance in lung carcinoma cells. A, Results of cytokine array (n = 1). Compared with the control, interleukin (IL)‐6 and IL‐8 protein expression was decreased in the miR‐1 transfected PC9 cells for 24 h. B, mRNA expression levels of cytokines (n = 3). C‐C Motif Chemokine Ligand 5 (CCL5) and C‐X‐C Motif Chemokine Ligand 10 (CXCL10) were significantly inhibited by miR‐1 transfection for 24 h in three cell lines (*p* < 0.05, see text for details). IL‐6, IL‐8, tumor necrosis factor (TNF)‐α were significantly inhibited in two cell lines other than PC9/ER (*p* < 0.05, see text for details). C, Transfection time was extended to 48 and 72 h in PC9/ER cell lines. IL‐6, IL‐8, TNF‐α were significantly inhibited (*p* < 0.05, see text for details) (n = 3). D, Representative findings of migrated monocytes (arrows) (bar: 100 µm). E, Monocyte migration was significantly inhibited by miR‐1 transfection (*p* = 0.0018) (n = 3)

We performed a cytokine array analysis using miR‐1 transfected PC9 for 24 h, against the control, in order to examine the effects of miR‐1 on the change of cytokine expression. Both IL‐6 and IL‐8 were decreased in the miR‐1 transfected cells (Figure 3A). CCL5, CXCL10, and TNF‐α, all of which were well known to be involved in immune cell migration, demonstrated no significant differences. Therefore, we subsequently validated the mRNA expression level of these cytokines (n = 3) and confirmed that those of CCL5 and CXCL10 were significantly inhibited by miR‐1 transfection for 24 h in these three cell lines above, PC9 (*p* = 0.0394, *p* = 0.0101, respectively), PC9/ER (*p* = 0.0015, *p* = 0.0072, respectively), H1975 (*p* = 0.0013, *p* = 0.0096, respectively), and those of IL‐6, IL‐8 and TNF‐α in two cell lines, PC9 (*p* = 0.0134, *p* = 0.0117, and *p* = 0.0047, respectively), H1975 (*p* = 0.0019, *p* < 0.001, and *p* = 0.0015, respectively) (Figure 3B). IL‐6, IL‐8 and TNF‐α were significantly decreased (48 h: *p* = 0.0008, *p* = 0.0195, *p* = 0.0006, 72 h: *p* = 0.0004, *p* = 0.0015, *p* = 0.0008, respectively) when the transfection time was extended to 48 and 72 h in PC9/ER cell lines (Figure 3C).

miRNAs have been reported to reduce the expression of cytokines involved in monocyte responses, including lymphocyte migration.^36^ Therefore, in this study, we performed a monocyte migration test in order to determine whether miR‐1 could inhibit the migration in human PBMC (n = 3). Representative findings are illustrated in Figure 3D. Monocyte migration was significantly inhibited in the miR‐1 transfected group (*p* = 0.0018) (Figure 3E). The migrated mononuclear cells were also confirmed under a light microscope and these cells were mainly composed of lymphocytes or macrophages.

## 4 DISCUSSION

In this study, we first examined the changes in the TIME before and after developing EGFR‐TKI resistance in human lung cancer tissues. Our results demonstrated that miR‐1 induction altered the sensitivity of EGFR‐TKI and the features of the TIME by inhibiting migration of monocytes, including CD8‐positive T cells. These results also provided new insights into the prediction of ICI efficacy and therapeutic strategy for lung cancer patients with EGFR‐TKI resistance.

In our present study, we focused on tumor‐infiltrating T lymphocytes as a factor influencing the TIME. This is because PD‐L1 has been widely used as a marker to predict the therapeutic efficacy of ICI, but the effects of ICI are known to be weak when tumor‐infiltrating T lymphocytes are relatively scarce, despite high levels of PD‐L1 in carcinoma cells.^37^, ^38^ In addition, in the cases whose levels of CD8‐positive T cell infiltration were high prior to EGFR‐TKI therapy, their levels decreased after acquiring EGFR‐TKI resistance. Although the number of cases was limited to only 21, the results of our immunohistochemical analysis was consistent with a previous study, in which 138 NSCLCs demonstrated that the status of CD8‐ and Foxp3‐positive T cells significantly decreased after developing EGFR‐TKI resistance.^26^ However, the mechanisms of the association between these changes of the TIME and EGFR‐TKI resistance has remained unknown. Therefore, we performed in vitro study to explore the rationale for reduction of lymphocytes infiltration after acquiring EGFR‐TKI resistance.

We first revealed an association between miR‐1 expression, TIME, and EGFR‐TKI resistance in lung adenocarcinoma patients. In particular, we focused on miRNAs because they are known to exert effects on various biological activities of cancer, such as progression and metastasis, and have recently been proposed as a useful marker to detect cancer status.^39^, ^40^, ^41^ We found that miR‐1 expression increased after acquiring EGFR‐TKI resistance in carcinoma cells although only one cell line model, revealed by comprehensive miRNA analyses. MiR‐1 has been found to play pivotal roles in muscle formation and muscle growth in skeletal and myocardial myocytes.^42^ It was subsequently reported to be involved in metastasis and suppression of progression in various human malignancies.^43^, ^44^, ^45^ On the other hand, there have been no reports regarding the interrelationship between miR‐1 and EGFR‐TKI resistance, to the best of our knowledge. Our results firstly clarified the significantly inhibitory effect of miR‐1 on EGFR‐TKI sensitivity. Although it is unclear whether an increase of miR‐1 is a cause or result of EGFR‐TKI resistance acquirement, we can consider that these results support the results of miRNA array analyses, and that an increase of miR‐1 is associated with EGFR‐TKI resistance in lung adenocarcinoma.

In addition, it is also true that miR‐1 expression was reported to be influenced by cytokines or other miRNAs involved in lymphocytes migration.^46^, ^47^, ^48^ However, it has been unknown whether miR‐1 regulates status of cytokines or lymphocyte migration or not. Results of our current comprehensive study of cytokines did reveal that the transfection of miR‐1 into three different human lung carcinoma cell lines harboring EGFR mutations significantly decreased the expression of CCL5, CXCL10, IL‐6, IL‐8, and TNF‐α. Among those cytokines, both CCL5 and CXCL10 are reported to be involved in the migration of CD8‐positive T cells and to induce migration of intratumoral CD8‐positive T cells in various tumors, including NSCLC.^49^, ^50^ In addition, both IL‐6 and IL‐8 were previously reported to be involved in the differentiation of CD8‐positive T cells and the migration of T cells.^51^, ^52^ Results of our present study reveal that miR‐1 transfection into human lung carcinoma cell lines harboring EGFR mutations does inhibit the migration of human monocytes. Therefore, we are able to demonstrate that miR‐1 reduced the expression of cytokines, resulting in an inhibition of monocytes, including lymphocyte migration. Unexpectedly, no significant reduction in cytokines was observed in PC9/ER compared with PC9/6M in this study (data not shown). However, as is well known, many factors have been known to regulate the expression of cytokines. Therefore, we thought that these unexpected results are because other factors affecting cytokines may have outweighed the effect of miR‐1 in PC9/ER. For further investigation to support our hypothesis that miR‐1 might suppress the TIME following development of EGFR‐TKI resistance, examination using multiple EGFR‐TKI resistant strains is required in the future.

In summary, the results of our present study indicate that intra‐tumoral CD8‐positive T cells are decreased in lung adenocarcinoma patients acquiring EGFR‐TKI resistance when levels of miR‐1 are raised. In addition, patients with high expression of miR‐1 may be less likely to benefit from subsequent ICI therapy. Therefore, an evaluation of serum miR‐1, before treatment and after acquiring EGFR‐TKI resistance, could contribute to predicting therapeutic efficacy of subsequent ICI therapy for the patient. In addition, chemotherapy rather than ICI treatment may be recommended for these patients, although further evaluation is required.

## CONFLICT OF INTEREST

All authors have no financial relationships to disclose.

## Funding information

This work was supported by JSPS KAKENHI Grant Numbers JP18 K15076.

## Data Availability

All data generated or analyzed during this study are included in this published article.

## References

[1] Bray F , Jemal A , Torre LA , Forman D , Vineis P . Long‐term realism and cost‐effectiveness: primary prevention in combatting cancer and associated inequalities worldwide. J Natl Cancer Inst. 2015;107(12):djv273. 26424777 10.1093/jnci/djv273 PMC4673394

[2] Bray F , Ferlay J , Soerjomataram I , Siegel RL , Torre LA , Jemal A . Global cancer statistics 2018: GLOBOCAN estimates of incidence and mortality worldwide for 36 cancers in 185 countries. CA Cancer J Clin. 2018;68(6):394‐424. 30207593 10.3322/caac.21492

[3] Oxnard GR , Binder A , Jänne PA . New targetable oncogenes in non‐small‐cell lung cancer. J Clin Oncol. 2013;31(8):1097‐1104. 23401445 10.1200/JCO.2012.42.9829 PMC3589703

[4] Kohno T , Tsuta K , Tsuchihara K , Nakaoku T , Yoh K , Goto K . RET fusion gene: translation to personalized lung cancer therapy. Cancer Sci. 2013;104(11):1396‐1400. 23991695 10.1111/cas.12275 PMC5439108

[5] Pao W , Hutchinson KE . Chipping away at the lung cancer genome. Nat Med. 2012;18(3):349‐351. 22395697 10.1038/nm.2697

[6] Kohno T , Nakaoku T , Tsuta K , et al. Beyond ALK‐RET, ROS1 and other oncogene fusions in lung cancer. Transl Lung Cancer Res. 2015;4(2):156‐164. 25870798 10.3978/j.issn.2218-6751.2014.11.11 PMC4384213

[7] Akamatsu H , Ninomiya K , Kenmotsu H , et al. The Japanese lung cancer society guideline for non‐small cell lung cancer, stage IV. Int J Clin Oncol. 2019;24(7):731‐770. 31049758 10.1007/s10147-019-01431-z PMC6545178

[8] Masters GA , Graham HF , Temin Jr S , et al. Systemic therapy for stage IV non – small‐cell lung cancer: American society of clinical oncology clinical practice guideline update. J Clin Oncol. 2015;33(30):3488‐3515. 26324367 10.1200/JCO.2015.62.1342 PMC5019421

[9] Van SPE , Hellmann MD , Peters S , Guidelines E . Metastatic non‐small cell lung cancer: ESMO clinical practice guidelines for diagnosis, treatment and follow‐up. Ann Oncol. 2018;29(Suppl 4):iv192‐iv237. 10.1093/annonc/mdy275 30285222

[10] Maemondo M , Inoue A , Kobayashi K , et al. North‐East Japan study group. Gefitinib or chemotherapy for non‐small‐cell lung cancer with mutated EGFR. N Engl J Med. 2010;362(25):2380‐2388. 20573926 10.1056/NEJMoa0909530

[11] Mitsudomi T , Morita S , Yatabe Y , et al. West Japan Oncology Group . Gefitinib versus cisplatin plus docetaxel in patients with non‐small‐cell lung cancer harbouring mutations of the epidermal growth factor receptor (WJTOG3405): an open label, randomised phase 3 trial. Lancet Oncol. 2010;11(2):121‐128. 20022809 10.1016/S1470-2045(09)70364-X

[12] Zhou C , Wu YL , Chen G , et al. Erlotinib versus chemotherapy as first‐line treatment for patients with advanced EGFR mutation‐positive non‐small‐cell lung cancer (OPTIMAL, CTONG‐0802): a multicentre, open‐label, randomised, phase 3 study. Lancet Oncol. 2011;12(8):735‐742. 21783417 10.1016/S1470-2045(11)70184-X

[13] Rosell R , Carcereny E , Gervais R , et al. Erlotinib versus standard chemotherapy as first‐line treatment for European patients with advanced EGFR mutation‐positive non‐small‐cell lung cancer (EURTAC): a multicentre, open‐label, randomised phase 3 trial. Lancet Oncol. 2012;13(3):239‐246. 22285168 10.1016/S1470-2045(11)70393-X

[14] Sequist LV , Yang JCH , Yamamoto N , et al. Phase III study of afatinib or cisplatin plus pemetrexed in patients with metastatic lung adenocarcinoma with EGFR mutations. J Clin Oncol. 2013;31(27):3327‐3334. 23816960 10.1200/JCO.2012.44.2806

[15] Yang JCH , Wu YL , Schuler M , et al. Afatinib versus cisplatin‐based chemotherapy for EGFR mutation‐positive lung adenocarcinoma (LUX‐Lung 3 and LUX‐Lung 6): Analysis of overall survival data from two randomised, phase 3 trials. Lancet Oncol. 2015;16(2):141‐151. 25589191 10.1016/S1470-2045(14)71173-8

[16] Park K , Tan EH , O’Byrne K , et al. Afatinib versus gefitinib as first‐line treatment of patients with EGFR mutation‐positive non‐small‐cell lung cancer (LUX‐Lung 7): a phase 2B, open‐label, randomised controlled trial. Lancet Oncol. 2016;17(5):577‐589. 27083334 10.1016/S1470-2045(16)30033-X

[17] Kobayashi S , Boggon TJ , Dayaram T , et al. EGFR mutation and resistance of non‐small‐cell lung cancer to gefitinib. N Engl J Med. 2005;352(8):786‐792. 15728811 10.1056/NEJMoa044238

[18] Cross DAE , Ashton SE , Ghiorghiu S , et al. AZD9291, an irreversible EGFR TKI, overcomes T790M‐mediated resistance to EGFR inhibitors in lung cancer. Cancer Discov. 2014;4(9):1046‐1061. 24893891 10.1158/2159-8290.CD-14-0337 PMC4315625

[19] Topalian SL , Hodi FS , Brahmer JR , et al. Safety, activity, and immune correlates of Anti–PD‐1 antibody in Cancer. N Engl J Med. 2012;366(26):2443‐2454. 22658127 10.1056/NEJMoa1200690 PMC3544539

[20] Brahmer J , Reckamp KL , Baas P , et al. Nivolumab versus docetaxel in advanced squamous‐cell non‐small‐cell lung cancer. N Engl J Med. 2015;373(2):123‐135. 26028407 10.1056/NEJMoa1504627 PMC4681400

[21] Borghaei H , Paz‐Ares L , Horn L , et al. Nivolumab versus docetaxel in advanced nonsquamous non‐small‐cell lung cancer. N Engl J Med. 2015;373(17):1627‐1639. 26412456 10.1056/NEJMoa1507643 PMC5705936

[22] Reck M , Rodriguez‐Abreu D , Robinson AG , et al. Pembrolizumab versus chemotherapy for PD‐L1‐positive non‐small‐cell lung cancer. N Engl J Med. 2016;375(19):1823‐1833. 27718847 10.1056/NEJMoa1606774

[23] Taube JM , Anders RA , Young GD , et al. Colocalization of inflammatory response with B7–h1 expression in human melanocytic lesions supports an adaptive resistance mechanism of immune escape. Sci Transl Med. 2012;4(127):127ra37. 10.1126/scitranslmed.3003689 PMC3568523 22461641

[24] Donnem T , Hald SM , Paulsen EE , et al. Stromal CD8+ T‐cell density ‐ a promising supplement to TNM staging in non‐small cell lung cancer. Clin Cancer Res. 2015;21(11):2635‐2643. 25680376 10.1158/1078-0432.CCR-14-1905

[25] Lee CK , Man J , Lord S , et al. Checkpoint inhibitors in metastatic EGFR‐mutated non‐small cell lung cancer—a meta‐analysis. J Thorac Oncol. 2017;12(2):403‐407. 27765535 10.1016/j.jtho.2016.10.007

[26] Isomoto K , Haratani K , Hayashi H , et al. Impact of EGFR ‐ TKI treatment on the tumor immune microenvironment in EGFR mutation – positive non – small cell lung cancer. Clin Cancer Res. 2020;26(8):2037‐2046. 31937613 10.1158/1078-0432.CCR-19-2027

[27] Haratani K , Hayashi H , Tanaka T , et al. Tumor immune microenvironment and nivolumab efficacy in EGFR mutation‐positive non‐small‐cell lung cancer based on T790M status after disease progression during EGFR‐TKI treatment. Ann Oncol. 2017;28(7):1532‐1539. 28407039 10.1093/annonc/mdx183

[28] Mascia F , Schloemann DT , Cataisson C , et al. Cell autonomous or systemic EGFR blockade alters the immune‐environment in squamous cell carcinomas. Int J Cancer. 2016;139(11):2593‐2597. 27509256 10.1002/ijc.30376 PMC5028304

[29] Lin K , Cheng J , Yang T , Li Y , Zhu B . EGFR‐TKI down‐regulates PD‐L1 in EGFR mutant NSCLC through inhibiting NF‐κB. Biochem Biophys Res Comm. 2015;463(1–2):95‐101. 25998384 10.1016/j.bbrc.2015.05.030

[30] Peng S , Wang R , Zhang X , et al. EGFR‐TKI resistance promotes immune escape in lung cancer via increased PD‐L1 expression. Mol Cancer. 2019;18(1):165. 31747941 10.1186/s12943-019-1073-4 PMC6864970

[31] Bartel DP . MicroRNAs: genomics, biogenesis, mechanism, and function. Cell. 2004;116(2):281‐297. 14744438 10.1016/s0092-8674(04)00045-5

[32] Lagos‐Quintana M , Rauhut R , Lendeckel W , Tuschl T . Identification of novel genes coding for small expressed RNAs. Science. 2001;294(5543):853‐858. 11679670 10.1126/science.1064921

[33] Weiss GJ , Bemis LT , Nakajima E , et al. EGFR regulation by microRNA in lung cancer: Correlation with clinical response and survival to gefitinib and EGFR expression in cell lines. Ann Oncol. 2008;19(6):1053‐1059. 18304967 10.1093/annonc/mdn006

[34] Hong L , Sharp T , Khorsand B , et al. MicroRNA‐200c represses IL‐6, IL‐8, and CCL‐5 expression and enhances osteogenic differentiation. PLoS One. 2016;11(8):1‐16. 10.1371/journal.pone.0160915 PMC4987006 27529418

[35] Nihira K , Miki Y , Iida S , et al. An activation of LC3A‐mediated autophagy contributes to de novo and acquired resistance to EGFR tyrosine kinase inhibitors in lung adenocarcinoma. J Pathol. 2014;234(2):277‐288. 24687913 10.1002/path.4354

[36] Garavelli S , De Rosa V , de Candia P . The multifaceted interface between cytokines and microRNAs: an ancient mechanism to regulate the good and the bad of inflammation. Front Immunol. 2018;9(December):3012. 30622533 10.3389/fimmu.2018.03012 PMC6308157

[37] Binnewies M , Roberts EW , Kersten K , et al. Understanding the tumor immune microenvironment (TIME) for effective therapy. Nat Med. 2018;24(5):541‐550. 29686425 10.1038/s41591-018-0014-x PMC5998822

[38] Forde PM , Chaft JE , Smith KN , et al. Neoadjuvant PD‐1 blockade in resectable lung cancer. N Engl J Med. 2018;378(21):1976‐1986. 29658848 10.1056/NEJMoa1716078 PMC6223617

[39] Lu J , Getz G , Miska EA , et al. MicroRNA expression profiles classify human cancers. Nature. 2005;435(7043):834‐838. 15944708 10.1038/nature03702

[40] Tomaru Y , Hayashizaki Y . Cancer research with non‐coding RNA. Cancer Sci. 2006;97(12):1285‐1290. 17052264 10.1111/j.1349-7006.2006.00337.x PMC11158021

[41] Calin GA , Croce CM . MicroRNA signatures in human cancers. Nat Rev Cancer. 2006;6(11):857‐866. 17060945 10.1038/nrc1997

[42] Wang XH . MicroRNA in myogenesis and muscle atrophy. Curr Opin Clin Nutr Metab Care. 2013;16(3):258‐266. 23449000 10.1097/MCO.0b013e32835f81b9 PMC3967234

[43] Karatas OF , Guzel E , Suer I , et al. miR‐1 and miR‐133b are differentially expressed in patients with recurrent prostate cancer. PLoS One. 2014;9(6):1‐7. 10.1371/journal.pone.0098675 PMC4072786 24967583

[44] Li D , Yang P , Li H , et al. MicroRNA‐1 inhibits proliferation of hepatocarcinoma cells by targeting endothelin‐1. Life Sci. 2012;91(11–12):440‐447. 22963810 10.1016/j.lfs.2012.08.015

[45] Letelier P , García P , Leal P , et al. MiR‐1 and miR‐145 act as tumor suppressor microRNAs in gallbladder cancer. Int J Clin Exp Pathol. 2014;7(5):1849‐1867. 24966896 PMC4069933

[46] Georgantas RW , Streicher K , Greenberg SA , et al. Inhibition of myogenic microRNAs 1, 133, and 206 by inflammatory cytokines links inflammation and muscle degeneration in adult inflammatory myopathies. Arthritis Rheumatol. 2014;66(4):1022‐1033. 24757153 10.1002/art.38292

[47] Simpson LJ , Ansel KM . MicroRNA regulation of lymphocyte tolerance and autoimmunity. J Clin Invest. 2015;125(6):2242‐2249. 26030228 10.1172/JCI78090 PMC4497751

[48] Swaminathan S , Suzuki K , Seddiki N , et al. Differential regulation of the let‐7 family of MicroRNAs in CD4 + T cells alters IL‐10 expression. J Immunol. 2012;188(12):6238‐6246. 22586040 10.4049/jimmunol.1101196

[49] Moran CJ , Arenberg DA , Huang CC , et al. Rantes expression is a predictor of survival in stage I lung adenocarcinoma. Clin Cancer Res. 2002;8(12):3803‐3812. 12473593

[50] Liu J , Li F , Ping Y , et al. Local production of the chemokines CCL5 and CXCL10 attracts CD8++ T lymphocytes into esophageal squamous cell carcinoma. Oncotarget. 2015;6(28):24978‐24989. 26317795 10.18632/oncotarget.4617 PMC4694808

[51] Yang R , Masters AR , Fortner KA , et al. IL‐6 IL‐6 promotes the differentiation of a subset of naive CD8+ T cells into IL‐21‐producing B helper CD8+ T cells. J Exp Med. 2016;213(11):2281‐2291. 27670591 10.1084/jem.20160417 PMC5068236

[52] Taub DD , Anver M , Oppenheim JJ , Longo DL , Murphy WJ . T lymphocyte recruitment by interleukin‐8 (IL‐8): IL‐8‐induced degranulation of neutrophils releases potent chemoattractants for human T lymphocytes both in vitro and in vivo. J Clin Invest. 1996;97(8):1931‐1941. 8621778 10.1172/JCI118625 PMC507263

